# Shrimps Down Under: Evolutionary Relationships of Subterranean Crustaceans from Western Australia (Decapoda: Atyidae: *Stygiocaris*)

**DOI:** 10.1371/journal.pone.0001618

**Published:** 2008-02-20

**Authors:** Timothy J. Page, William F. Humphreys, Jane M. Hughes

**Affiliations:** 1 Australian Rivers Institute, Griffith University, Nathan, Queensland, Australia; 2 Western Australian Museum, Welshpool DC, Western Australia, Australia; University of Dayton, United States of America

## Abstract

**Background:**

We investigated the large and small scale evolutionary relationships of the endemic Western Australian subterranean shrimp genus *Stygiocaris* (Atyidae) using nuclear and mitochondrial genes. *Stygiocaris* is part of the unique cave biota of the coastal, anchialine, limestones of the Cape Range and Barrow Island, most of whose nearest evolutionary relations are found in coastal caves of the distant North Atlantic. The dominance of atyids in tropical waters and their food resources suggest they are pivotal in understanding these groundwater ecosystems.

**Methodology/Principle Findings:**

Our nuclear and mitochondrial analyses all recovered the Mexican cave genus *Typhlatya* as the sister taxon of *Stygiocaris*, rather than any of the numerous surface and cave atyids from Australia or the Indo-Pacific region. The two described *Stygiocaris* species were recovered as monophyletic, and a third, cryptic, species was discovered at a single site, which has very different physiochemical properties from the sites hosting the two described species.

**Conclusions/Significance:**

Our findings suggest that *Stygiocaris* and *Typhlatya* may descend from a common ancestor that lived in the coastal marine habitat of the ancient Tethys Sea, and were subsequently separated by plate tectonic movements. This vicariant process is commonly thought to explain the many disjunct anchialine faunas, but has rarely been demonstrated using phylogenetic techniques. The Cape Range's geological dynamism, which is probably responsible for the speciation of the various *Stygiocaris* species, has also led to geographic population structure within species. In particular, *Stygiocaris lancifera* is split into northern and southern groups, which correspond to population splits within other sympatric subterranean taxa.

## Introduction

By their very nature, caves and other subterranean environments are poorly known, and yet they are home to large numbers of endemic species and unique relictual taxa from an earlier age [1] (“wrecks of ancient life” in the words of Charles Darwin [2]). The various evolutionary and geographic patterns displayed by subterranean biota can illuminate the complex processes and histories that have resulted in this biodiversity [3], [4], in much the same way as has been demonstrated for isolated oceanic islands [5]. The isolation and strong selective pressures inherent in the adoption of an underground life can lead in polar opposite directions, namely both genetic divergence and morphological convergence [6], which can greatly confound interpretation [7]–[9]. Darkness, low energy inputs and many other common factors in subterranean environments often lead to very different animals evolving similar traits, such as atrophied eyes and a translucent body [10]. Once these “troglomorphies” have arisen, a species is presumed to have a limited dispersal ability owing to its highly structured and isolated environment and the very narrow range of habitat to which it is adapted [7], [11].

**Figure 1:**
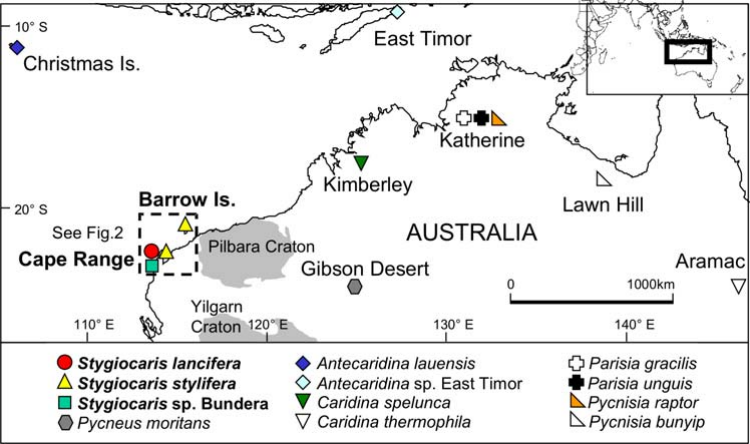
Map of collection locations of subterranean atyids in the Australian region. See Tables 1, 2 for site details.

Australia was once thought to have few areas of interest for those with a bent towards the underworld [1], [12], but this has changed dramatically in recent decades, particularly in Western Australia [1], [10], [13]. The Precambrian landscape of inland Western Australia's Pilbara and Yilgarn Cratons (the “Western Shield”, Fig. 1) is one of the oldest and most stable on earth [14]. Beneath this parched, ancient landscape, many independent “calcrete” (terrestrial limestone) aquifers have formed by precipitation of thin layers of carbonates along old drainage lines [14]. A number of molecular studies of various taxa (diving beetles, amphipods, bathynellacea [5], [6], [15], [16]) of this “subterranean archipelago” [5] have confirmed the heterogeneous evolutionary origin of its endemic fauna and the isolated island nature of the environment referred to above.

**Figure 2:**
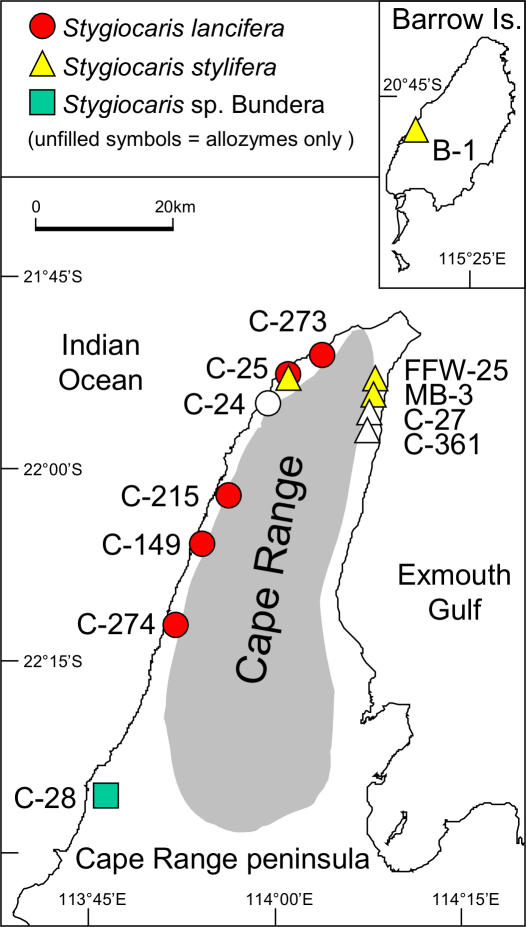
Map of *Stygiocaris* collection locations in the Cape Range peninsula and Barrow Island, northwestern Australia. See Tables 1 for site details.

A highly diverse and distinct subterranean fauna is also found on the western edge of the Pilbara Craton, in the younger limestones of the Cape Range peninsula and Barrow Island (Fig. 2) [17]. The evolutionary relationships of this biota have not yet been studied using DNA sequences, but earlier small-scale studies have used allozymes [18]–[20] and found the presence of cryptic species and localised geographic structuring. In some ways, the Cape Range area is potentially even more interesting than the calcretes of the Western Shield, because its flooded coastal limestone caves and fissures (karst) are rare in Australia, and because, in contrast to the geological stability of the Western Shield, the Cape Range area has been geologically dynamic [1], [21], which can lead to high levels of biodiversity [4].

Today a low, arid, mountain range (∼300 m high) runs north-south along the spine of the tropical Cape Range peninsula (80 kms by 20 kms) (Fig. 2), fringed on the western and northern sides by the Ningaloo Coral Reef. Until the Tertiary, this whole area was covered with a warm, shallow sea in which limestones were formed against the coastline of the Western Shield [19]. Upfolding of the limestones in the Miocene formed a series of anticlines, two of which comprise the Cape Range, originally possibly an island [21]–[23], and Barrow Island, 170 km to the northeast (Fig. 2) [24]. Exposure of the raised limestone to solution by mildly acidic rainwater formed caves and gorges, characteristic of karst terrain [3], [25]. This was especially so in the middle of the limestone sequences, the Tulki Limestone, that is now highly cavernous [23]. Lower sea levels during the Pleistocene (50–150 m lower than present) would have exposed a 12 km wide plain to the west of Cape Range, and a continuous plain between northern Cape Range and Barrow Island. This plain was intersected by several rivers (Ashburton, Cane, Robe) which were similar in nature to those bordering the Cape Range peninsula today [18].

**Figure 3:**
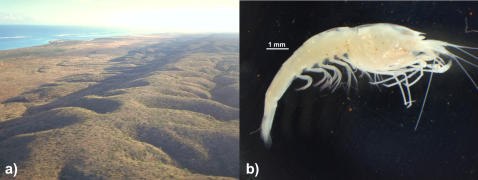
Photos of a) Cape Range and b) *Stygiocaris stylifera*. Aerial photo by WFH, and shrimp photo courtesy of Dr. Danny Tang.

The subterranean habitats of the Cape Range area are largely of two types [17]. Firstly, there are those within the range itself, which are either dry or have small perched aquifers [19]. The biota here is relictual, largely related to distant Australian terrestrial humid-forest species which probably retreated underground as the climate became arid in the Miocene [17], [20], as did the fauna of the Western Shield [15]. The second habitat is an extensive flooded underground karst wetland, found on the narrow, flat coastal plain (up to 2 km wide; Fig. 3a) of the peninsula and extending under the foothills. This habitat also occurs on Barrow Island [18]. This coastal fauna is aquatic and unrelated to that of the range [18], and elements of it also occur on the poorly known Pilbara coastal strip to the north-east [24], [26]. The coastal aquifer is in the form of a wedge of sea water which intrudes beneath freshwater. This aquifer probably varies with location (major gorges discharge to the coast [18], [21]) and with the episodic recharge of the aquifer in this region affected by tropical cyclones [18], [27]. In consequence, salinity levels tend to be lower at the top of the water column and with increasing distance from the coast but vary between freshwater (cave C-215) to full seawater (deeper parts of Bundera Sinkhole, C-28). Tidal fluctuation in the groundwater progressively decreases inland of the coast but may still be 10% of ocean tides 1.7 km inland [28].

Coastal waterbodies with subterranean connections to the sea and tidal influences are known as “anchialine” [25], and are found throughout the tropics and sub-tropics, often on arid coasts [10]. These habitats are characterised by variable salinity and light, and can be thought of as “groundwater estuaries” [10]. The fauna found in anchialine habitats are often highly disjunct, relictual taxa, often thought to descend from shallow marine populations of the Tethys Sea, which once separated the ancient supercontinents of Gondwana and Laurasia [25], [29], [30], implying vicariance by continental drift. The Tethyan Seaway between the Mediterranean and Arabian Seas allowed frequent marine faunal interchange between the Atlantic and Indo-West Pacific regions until at least 19 million years ago [31], and possibly as recently as 10 million years ago [32].

The coastal plain habitat of Cape Range supports a fauna of a type unknown elsewhere in the southern hemisphere, also occurring in similar anchialine habitats on either side of the North Atlantic (Yucatán, Mexico, islands of northern Caribbean, and Canary Islands). Although most of this fauna is known only in Australia from the Bundera Sinkhole, the sole accessible deep anchialine system, elements of the fauna occur widely in the more superficial waters of the linear Cape Range coastal plain, Barrow Island and the Pilbara coast (e.g. *Stygiocaris* Holthuis, 1960 (Atyidae), *Halosbaena* Stock, 1976 (Thermosbaenacea), *Haptolana* Bowman, 1966 (Cirolanidae), various amphipods [17]–[19], [27], and Australia's only subterranean fishes, a blind eel and gudgeon [26]). In contrast to the origins of the taxa from both the Cape Range itself and from the Western Shield, the evolutionary origins of the taxa from the coastal plain habitat are more obscure and likely to be marine.

Crustaceans are common in subterranean habitats [8], [10], [33], and shrimps from the family Atyidae are frequently found in both major types of anchialine habitat, namely those of continental coasts (“remipede-type”) and seamount islands (“procarid-type”) [29], [34]. The Atyidae are an ancient group of decapod shrimps comprising dozens of genera spread throughout the globe and largely occurring in freshwaters [35]. Although many atyid species have brackish water larval phases [34], there are no known extant marine relatives [36], which is a common situation for anchialine species [4], [25], [37].

The Cape Range coastal plain and Barrow Island host two endemic species of atyid shrimp, *Stygiocaris lancifera* Holthuis, 1960 and *S. stylifera* Holthuis, 1960 (Fig. 3b). *Stygiocaris* is potentially an ideal window into the evolution and biogeography of this biota because 1) it is widespread within the coastal plain and Barrow Island [27], 2) the two species are largely separated east and west by the range (Fig. 2), and so small scale microevolutionary and phylogeographic patterns within the area may be discernible, and 3) large-scale evolutionary and biogeographic patterns (which are rarely studied for groundwater species [4]) may also be reconstructed because, although *Stygiocaris* is endemic to the area, its hypothesized closest evolutionary relatives are all geographically remote, namely *Typhlopatsa* Holthuis, 1956 from Madagascar [38] and other genera from the atyid sub-family Typhlatyinae [25], [39] (*Antecaridina* Edmondson, 1954: Indo-Pacific [36]; *Typhlatya* Creaser, 1936: Caribbean, Europe [30], [40]).

Molecular phylogenetic and clock analyses can help to cut through the confusion of morphological convergence to help to resolve the evolutionary and biogeographic history of subterranean taxa [41]–[43]. As subterranean atyids have been the subject of a number of molecular studies in Europe [44], the Caribbean [40], [45], the Pacific [46], [47] and Australia [35], as have surface atyids (e.g. [48]–[50]), there is an excellent context within which to place new data to test evolutionary hypotheses of the Western Australian stygobites.

Previous studies of Australian [35] and European atyid [44] genera have found that subterranean species are often related to local surface species rather than other distant “congeneric” cave dwellers. Molecular studies of anchialine atyids have inferred at least occasional marine dispersal within an evolutionary timeframe [40], [46], [47] and a study of an anchialine snail found large amounts of recent gene flow over large ranges (>200 km) [51]. This implies that an ancient “Tethyan” vicariant explanation may not be required to explain the presence of *Stygiocaris* in northwestern Australia. Therefore, we hypothesized that *Stygiocaris* will be an evolutionary offshoot of one of the many surface or anchialine species of the Indo-Pacific or Australia, particularly as coastal Western Australia hosts many undescribed surface atyids ([48] and TJP unpublished data). At the smaller scale, we hypothesized that the geological dynamism of the area will be reflected in the local structuring of *Stygiocaris* populations into isolated, geographically distinct biological units, much as it has done for other groundwater fauna of the area [19].

Dense populations of *Stygiocaris* (138 m^2^) have been recorded in favourable caves [52] and they access a wide range of food resources [24]. Atyids are a major component of many surface waters [53], where they consume fine organic matter and biofilms [54]. These are characteristic energy sources in groundwater, and so *Stygiocaris* are expected to be similarly pivotal to understanding the dynamics of the anchialine system.

## Methods

### Specimen collection

We collected specimens of the *Stygiocaris* species from throughout their distributions on the Cape Range peninsula and Barrow Island, Western Australia (Fig. 2, Table 1). Specimens were collected at various sites using a number of methods. We used hand or plankton haul nets (mesh size 125–350 µm) within caves, historical pastoral wells, oil field anode protection bores, water supply and monitoring bores in town, and Defence establishment water supply aquifers. Within the Bundera Sinkhole (an anchialine cave [28]), cave divers towed a net below the hydrogen sulphide layers. As these sites are remote and difficult to access, collection numbers of *Stygiocaris* are generally small [52] and only a limited number could be analysed per site (Table 1).

**Table 1:** Western Australian Stygiocaris specimens and sequences.

Species	Site Code	Site Name	Voucher Numbers	Latitude (S)	Longitude (E)	16S (specimen *N*)	COI (specimen *N*)	Histone (specimen *N*)
*Stygiocaris lancifera*	C-273	Five Mile Well*	BES693	21.850	114.065	EU123831(1)		
	C-25	Kuddamurra Well*	BES9733-6, 9743	21.888	114.009	EU123831(3),EU123832(1), EU123834(1)	EU123825(1),EU123826 (1)	EU123805(1)
	C-215	unnamed cave*	BES2205, 9783-7	22.028	113.932	EU123828(3),EU123830(2), EU123833(1)	EU123824(1)	
	C-149	Tulki Well*	BES9788-92	22.092	113.897	EU123827(2),EU123828(1), EU123829(1),EU123830(1)	EU123823(1)	EU123804(1)
	C-274	Pilgonoman Well*	BES670	22.192	113.866	EU123827(1)		
*Stygiocaris stylifera*	B-1	Ledge Cave#	BES3376	20.798	115.331	EU123837(1)	EU123815(1)	EU123806(1)
	FFW-25	Defense Bore*	BES14066	21.894	114.101	EU123835(1)		
	MB-3	Defense Bore*	BES14068	21.908	114.098	EU123836(1)	EU123814(1)	EU123806(1)
	C-25	Kuddamurra Well*	BES9737-41, 9745-7	21.888	114.009	EU123838(2),EU123839(6)	EU123816(1),EU123817 (1),EU123818(2)	EU123806(1)
*Stygiocaris* sp. Bundera	C-28	Bundera Sinkhole*	BES3477, 3562, 3950,3952,3954, 4711,4722	22.414	113.764	EU123840(5),EU123841(1), EU123842(1)	EU123819(3),EU123820 (2),EU123821(1)	EU123807(2)
Outgroup								
*Antecaridina* sp. East Timor		Umun Ira, East Timor	GU-1122	8.354	127.051	EU123853(1)	EU123822(1)	EU123813(1)

* = Cape Range; # = Barrow Island; BES = Biospeleology, Western Australian Museum; GU = Griffith University.

All specimens from Western Australian Museum except *Antecaridina* sp. East Timor from John Short (BioAccess).

For context, we also included specimens of all the known subterranean atyid shrimp species in Australia (Fig. 1, Table 2). A number of these taxa have not been previously sequenced, including the Western Australian genus *Pycneus* Holthuis, 1986 (Gibson Desert), the Western Australian species *Caridina spelunca* Choy, 1996, and the Northern Territory species *Parisia gracilis* Williams, 1964. Also included were a number of anchialine species from the Indo-Pacific (*Antecaridina* spp., *Halocaridina* Holthuis, 1963), and various epigean and subterranean species from throughout the world. Where possible, we integrated published sequences of numerous atyid species into our datasets (Table 2). Specimens of various taxa were kindly provided to us by many museums, institutions and individuals (Tables 1, 2).

**Table 2:** Additional specimens and sequences included in Worldwide 16S and H3 analyses.

Genus	Species	Sample Site^Specimen Provenance^	GenBank Accession Numbers	
			16S	Histone
*Antecaridina*	*lauensis * #	19th Hole Cave, Christmas Island^A^	EU123851, EU123852	EU123812
	*lauensis* #	Whip Cave, Christmas Island^A^	EU123850	
	sp. East Timor #	Umun Ira, East Timor^B^	EU123853	EU123813
*Atyaephyra*	*desmarestii*	Al-Huaizah marshes, Iraq^C^	EU123848	
*Atyoida*	*bisulcata*	Hawaii*		DQ079661*
*Australatya*	*striolata*	Johnstons Ck., NSW, Australia^D^	AY795035*	
*Caridina*	*africana*	Hayfields, Pietermaritzburg, South Africa^E^	DQ478483*	
	*confusa*	Molo Ck., QLD, Australia^F^	DQ478450*	
	*indistincta* C4	Byron Ck., NSW, Australia^D^	AY795049*	
	sp. LE	Algebuckina Waterhole, Neales R., SA, Australia^G^	DQ478534*	EU123809
	sp. NT 1	Melville Is., NT, Australia^H^	DQ478537*	
	sp. WA 2	Camp Ck., King Edward R., WA, Australia^I^	DQ478550*	
	sp. WA 3	Gnieraoora Pool, Onslow Coast, WA, Australia^I^	DQ478552*	
	sp. WA 4	Mantinea Flats, Ord R., WA, Australia^I^	DQ478555*	
	*spelunca* #	Old Napier Downs Cave, Kimberleys, WA, Australia^A^	EU123845	
	*spelunca* (sp. WA1)	Anne Ck., Lennard R., WA, Australia^I^	DQ478549*	
	*spinula*	McIlwraith Range, Lockart, QLD, Australia^J^	DQ478527*	
	*steineri * #	Lakata Zafera, Madagascar^J^	DQ681249*	
	*thermophila * #	Aramac, QLD, Australia^K^	EU123846	
	*zebra*	Davidson Ck., Tully, QLD, Australia^L^	AY661486*	
*Halocaridina*	*rubra * #	Halape Iki, Hawaii^M^	EF490008*	EU123808
*Paratya*	*australiensis*	Lake Crescent, TAS, Australia^D^	DQ478566*	
	*curvirostris*	Marawara Stream, Waitakere Ranges, New Zealand^N^	AY661476*	
*Parisia*	*gracilis * #	Cutta Cutta Caves, Katherine, NT, Australia^O^	EU123843, EU123844	EU123810
	*unguis * #	Cutta Cutta Caves, Katherine, NT, Australia^A^	DQ681289*	
*Pycneus*	*morsitans * #	Mujingerra Cave, Gibson Desert, WA, Australia^A^	EU123849	EU123811
*Pycnisia*	*bunyip * #	Forbes Inferno Cave, Riversleigh, QLD, Australia^F^	N/A	
	*raptor * #	Grants Cave, Katherine, NT, Australia^O^	DQ681271*	
*Spelaeocaris*	*pretneri * #	Ljelješnica, Dabarsko polje, Bosnia*	DQ641590*	
*Troglocaris*	*anophthalmus * #	Kačna jama, cave, Divača, Slovenia*	DQ641571*	
*Typhlatya*	*pearsi * #	Cenote Santa Maria, Yucatán Peninsula, México*	AY115539*	DQ079702*
Outgroups				
*Macrobrachium*	*australiense*	Dimond Gorge, Fitzroy R., WA, Australia^I^	EF588317*	
	*potuina*	the Americas*	AY377851*	DQ079685*
*Metapenaeus*	sp.	Baffle Ck., QLD, Australia^D^	EU123847	

# = Subterranean;

* = sequence from GenBank; NSW = New South Wales; NT = Northern Territory; QLD = Queensland; SA = South Australia; TAS = Tasmania; WA = Western Australia.

Specimen sources: ^A^WA Museum; ^B^J.Short; ^C^M.Nasser; ^D^Griffith University; ^E^R.Hart; ^F^QLD Museum; ^G^S.Barter; ^H^SA Museum;^ I^M.Scanlon; ^J^S.Choy; ^K^R.Smith; ^L^D.Hurwood; ^M^K.Hopkins; ^N^K.Collier; ^O^NT Museum.

### Laboratory

Genomic DNA was extracted, amplified and sequenced as per [48]. Two mitochondrial genes and one nuclear gene were targeted. The mitochondrial large subunit 16S ribosomal DNA (16S) was chosen because it is effective for both higher and lower systematic level phylogenetics [33] and is the best represented gene on GenBank for the Atyidae (August 2007). 16S was sequenced for all specimens as per [48] (Tables 1 and 2 for all GenBank accession numbers). We also sequenced a subset of the *Stygiocaris* specimens for the more quickly-evolving mitochondrial gene cytochrome oxidase subunit I (COI), which is effective at discriminating at the population and species-level [33], and is the locus favoured in the push for “DNA barcoding” (see [43]). For the COI amplification, we used primers CR-COI-F (5′-CWA CMA AYC ATA AGA YAT TGG-3′) and CR-COI-R (5′-GCR GAN GTR AAR TAR GCT CG-3′) [55]. For a conserved nuclear gene, we sequenced Histone (H3) as per [56], who show it is informative for deep-level decapod phylogenetics. We sequenced the H3 gene for *Stygiocaris* spp. and any atyid species that fell within a higher-level 16S clade with it.

### Datasets

We assembled a number of separate datasets with various combinations of genes and taxa to investigate different phylogenetic levels (Table 3). Sequences were trimmed separately for each dataset so sequences for all terminal taxa were the same length. Two of the datasets (“Atyid 16S”, “*Stygiocaris* 16S”) include only 16S sequences. The Atyid 16S dataset includes 13 genera of atyids (10 of which have subterranean species) from throughout the world, and includes all eight genera found in Australia. The *Stygiocaris* 16S dataset includes sequences from all of our specimens of *Stygiocaris* from nine sampling sites. The 16S sequences from these two datasets were aligned using ClustalX version 1.81 [57] at default settings, and Gblocks version 0.91b [58] was used to identify poorly aligned sites, which were excluded from analyses. The sequences from the remaining datasets were aligned with ClustalX as above, with no sites excluded.

**Table 3:** Different datasets, molecular models and tree scores for analyses conducted in this study.

Dataset	Genes	Fig.	Molecular models from Modeltest	Tree Scores		
				ML	Bayesian	Parsimony (steps)
Atyid 16S	16S	4	HKY+I+G	–4750.25	–4750.85	982
Histone	H3	5	TrNef+I+G	–1248.99	–1283.85	183
Combined 16S/H3	16S/H3	N/A	GTR+I+G (combined), TIM+I+G (16S), TrNef+I+G (H3)	–4215.37	–4122.84	714
*Stygiocaris* 16S	16S	6	HKY+G	–1839.58	–1867.00	253
*Stygiocaris* Combined	H3/16S/COI	7	GTR+G (combined), TrNef (H3), K81uf+I (16S), GTR+G (COI)	–4055.79	–4000.30	484

ML = maximum likelihood; GTR =  General Time Reversible; HKY =  Hasegawa-Kishino-Yano; K81uf =  Kimura 3-parameter unequal-frequency; TIM =  Transition; TrNef =  Tamura-Nei equal-frequency; +I =  proportion of invariable sites; +G =  gamma distribution of site-to site variation.

The “Histone” dataset incorporates conserved nuclear H3 sequences from eight atyid genera, which were also analysed in combination with the relevant 16S sequence (“Combined 16S/H3”). “*Stygiocaris* Combined” includes H3, 16S and COI sequences from the three *Stygiocaris* species (and major intraspecific groupings) with *Antecaridina* sp. East Timor as an outgroup. Each gene region was analysed separately by gene as well as combined. We aligned H3 and COI sequences without gaps. Two further datasets of all available *Stygiocaris* 16S and *Stygiocaris* COI sequences respectively were also assembled to create haplotype networks and to derive genetic divergence estimates within and between species.

### Analyses

We used Modeltest version 3.06 [59] to select the Akaike Information Criterion best-fit model of evolution for each dataset separately. For the *Stygiocaris* Combined and Combined 16S/H3 Datasets, an appropriate model was selected for each gene, as well as for all genes combined. Three forms of phylogenetic analysis were employed. We used PHYML version 2.4.4 [60] for maximum likelihood analyses, MrBayes version 3.1.2 [61] for Bayesian analyses (parameters: 2 million generations, trees sampled every 100 cycles, datasets partitioned by gene where appropriate, 50% burn in, two runs of four chains heated to 0.2), and PAUP* version 4.0 b10 [62] for parsimony analyses (full heuristic with 100 random repetitions). Maximum likelihood and parsimony analyses were bootstrapped 1000 times.

Phylogenetic hypotheses of the sister taxon of *Stygiocaris* in the Combined 16S/H3 dataset were investigated using the Shimodaira–Hasegawa (S–H) test in PAUP (1000 replicates of resampling of estimated log-likelihood test distribution) and Bayes Factors [63] in MrBayes (constrained versus unconstrained harmonic means of likelihood values [64]).

Haplotype networks were constructed separately for all *Stygiocaris* 16S and COI sequences using TCS version 1.21 [65]. COI and 16S sequence divergences within and between *Stygiocaris* species were calculated using a correction for within-group polymorphism [66] (and ±S.E.). Distance matrices were constructed in PAUP* using both uncorrected and corrected divergences (using the suggested models of molecular evolution from Modeltest).

### Molecular clock calculations

Because of the geological dynamism of the Cape Range area, there is likely a close relationship between geological and biological events [17], [21]. If we accept the likelihood that the emergence of the Cape Range Anticline in the Miocene isolated the ancestors of *Stygiocaris lancifera* and *S. stylifera*, leading to their speciation [17], then we can use this event as a calibration point to estimate rates of molecular divergence for these taxa. This geological event is dated to the Miocene 7–10 million years ago (MYA) [21], [22].

As molecular clock calculations are often contentious, in particular for cave species [67], we used two methods of calculating molecular divergence rates. Firstly, we used a simple distance method, using the various COI and 16S sequence divergences between *S. lancifera* and *S. stylifera* referred to just above. We assumed the *S. lancifera*/*stylifera* split occurred 7–10 MYA and applied the derived rates from this single node to the divergence between *S. lancifera*/*stylifera* and *S*. sp. Bundera. Secondly, we used a relaxed (uncorrelated lognormal) molecular clock method [68] as implemented in BEAST version 1.4.6 [69]. We did two independent runs of BEAST (chain length of 10,000,000; sampled every 1000; Yule speciation process; 10% burn in) and combined the results with Tracer version 1.4 [70] to calculate the time to most recent common ancestor (tmrca) for two nodes, *Typhlatya/Stygiocaris* spp. and *S. lancifera*/*stylifera*/sp. Bundera, by fixing the *S. lancifera*/*stylifera* tmrca to fall within the 7–10 million year range.

## Results

*Pycnisia bunyip* Suzuki & Davie, 2003 did not produce usable sequences. The Modeltest-derived models and tree scores from maximum likelihood, Bayesian and parsimony analyses for all phylogenetic datasets appear in Table 3.

### Higher-level relationships of Australian subterranean species

**Figure 4:**
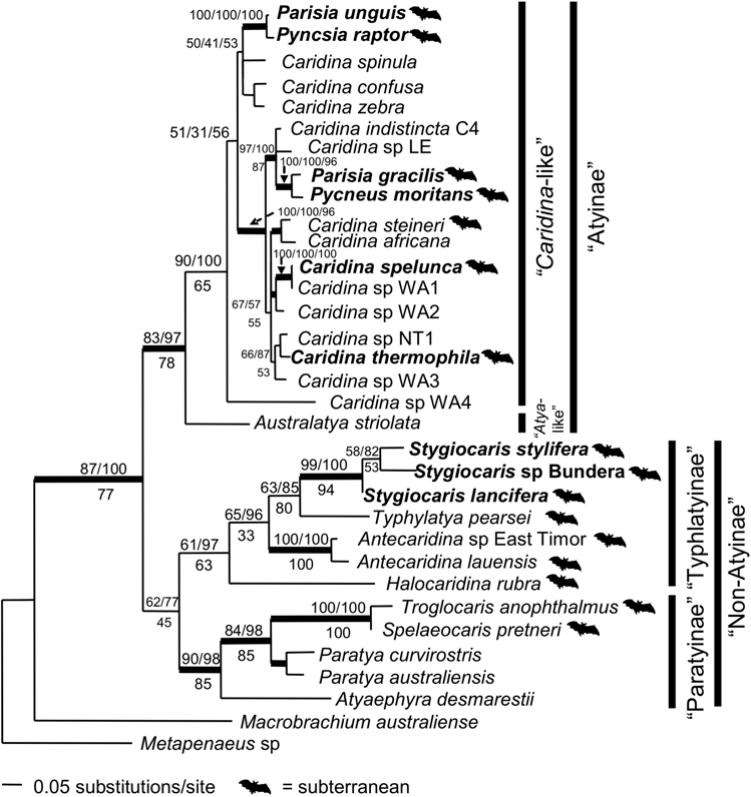
Maximum likelihood phylogram of mitochondrial Atyid 16S dataset. Australian subterranean species in bold. Branches with support >75% for all forms of analysis have thicker lines (Maximum Likelihood bootstrap values/Bayesian posterior probabilities above node, and Parsimony bootstrap values below).

At the largest phylogenetic scale (Fig. 4), the atyids fell into two higher-level clades, the “Atyinae” and “non-Atyinae” (*sensu* [35], [39]). Australian subterranean species were found within both groups. There were three distinct taxa within *Stygiocaris*, implying the presence of a cryptic species (*Stygiocaris* sp. Bundera). The sister to *Stygiocaris* was the Mexican cave shrimp *Typhlatya pearsei* Creaser, 1936. The anchialine *Halocaridina* and *Antecaridina* (which also has an undescribed species) were recovered within a clade containing both *Stygiocaris* and *Typhlatya*. Interestingly, all of the species in this “Typhlatyinae” clade (*sensu* [35], [39]) (Fig. 4) have subterranean proclivities. The other major clade within the Non-Atyinae (“Paratyinae” *sensu* [35], [39]) has both cave and surface species, although, interestingly, both of these two surface species of *Paratya* Miers, 1882 have also been reported from caves [12], [71].

All other Australian subterranean species fall within the “*Caridina*-like” group. As previously found [35], the cave species *Parisia unguis* Williams, 1964 and *Pycnisia raptor* Bruce, 1992 form a strong clade. Similarly, the other species of *Parisia* Holthuis, 1956 in Australia, *Parisia gracilis*, forms a clade with another cave genus, *Pycneus*, but both sets of clades group with completely separate surface species of *Caridina* H. Milne Edwards, 1837. This implies an independent colonisation of the underworld in Australia and morphological convergence. The DNA sequence of the Western Australian cave species, *Caridina spelunca*, is nearly identical to a previously unidentified surface species from the same area (*Caridina* sp. WA1; [48]), implying that these are conspecific and that *Caridina spelunca* is a troglophile, namely, a facultative subterranean inhabitant (as suggested by [72]).

**Figure 5:**
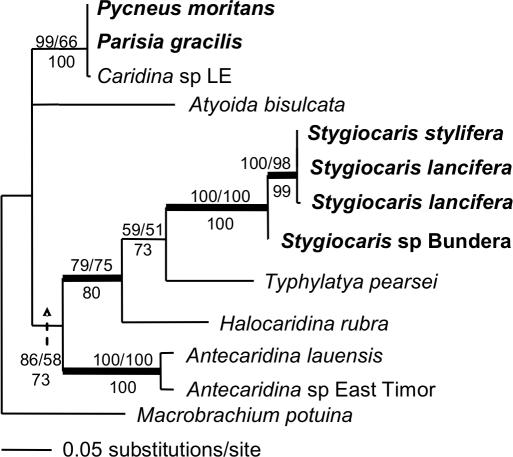
Maximum likelihood phylogram of nuclear Histone dataset. Australian subterranean species in bold. Branches with support >75% for all forms of analysis have thicker lines (Maximum Likelihood bootstrap values/Bayesian posterior probabilities above node, and Parsimony bootstrap values below).

Alternative explanations for some of these mitochondrial relationships are long-branch attraction and mitochondrial introgression [43], and so we also sequenced the highly conserved nuclear Histone (H3) gene for a sub-set of taxa. This nuclear Histone dataset (Fig. 5) recovered very similar relationships to the larger mitochondrial 16S dataset (Fig. 4), which implies that the evolutionary relationships recovered with 16S may accurately reflect species history, and not merely the organelle history. *Stygiocaris stylifera* and *S. lancifera* are closely related (sharing a haplotype) relative to *Stygiocaris* sp. Bundera, which is distinct. In this nuclear dataset, *Typhlatya* was again recovered as the sister to *Stygiocaris*, and both again formed a clade with *Halocaridina* and *Antecaridina*. As in the 16S dataset, *Parisia gracilis*, *Pycneus* and *Caridina* sp. LE are very closely related (with *P. gracilis* and *Pycneus* sharing an H3 haplotype). When the H3 and 16S sequences were combined and analysed as above (not displayed), the topology was identical to the H3-only dataset (Fig. 5). Support values for the combined H3/16S sequences were higher than in the analyses of H3-only for the *Stygiocaris/Typhlatya* relationship (Maximum Likelihood/Bayesian/Parsimony: 78/79/75), and comparable for the *Stygiocaris/Typhlatya/Halocaridina* relationship (66/75/88).

Every best-scoring topology from all three forms of analysis of the three relevant datasets (Atyid 16S, Histone, Combined 16S/H3) recovered *Typhlatya* as the sister taxon to *Stygiocaris*. But, as *Halocaridina* or *Antecaridina* spp. are also potential sisters of *Stygiocaris* in place of *Typhlatya* (see Figs. 4, 5), we calculated tree likelihoods for topologies constrained to either *Halocaridina* or *Antecaridina* as a sister to *Stygiocaris*. We then compared them to unconstrained trees (in which *Typhlatya* was always sister to *Stygiocaris*). Using Bayes Factors, the evidence against a hypothesis of *Halocaridina* as a sister was “Substantial” ([63]; 2×differences in logs = 6.12) (unconstrained harmonic mean marginal likelihood = –4122.84; *Halocaridina* constrained likelihood = –4125.90). The evidence against an *Antecaridina* sister was “Very Strong” (2×logs = 15.10) (*Antecaridina* constrained likelihood = –4130.39). The S-H test could not reject either *Halocaridina* or *Antecaridina* as potential sisters to *Stygiocaris* at the 0.05 level but would reject them both at the 0.10 level (*Halocaridina P* = 0.069; *Antecaridina P* = 0.085).

### *Stygiocaris* species

**Figure 6:**
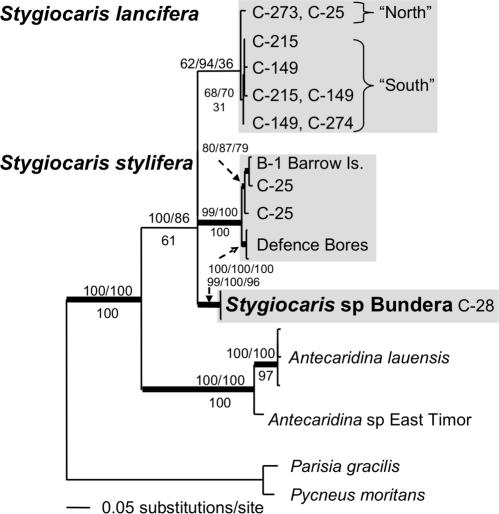
Maximum likelihood phylogram of *Stygiocaris* 16S dataset. Branches with support >75% for all forms of analysis have thicker lines (Maximum Likelihood bootstrap values/Bayesian posterior probabilities above node, and Parsimony bootstrap values below).

**Figure 7:**
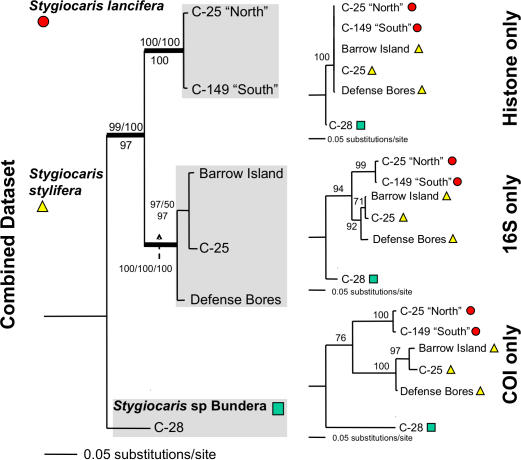
Maximum likelihood phylogram of *Stygiocaris* Combined dataset (H3/16S/COI). Branches with support >75% for all forms of analysis have thicker lines (Maximum Likelihood bootstrap values/Bayesian posterior probabilities above node, and Parsimony bootstrap values below). Also, individual gene maximum likelihood phylograms. Outgroup (*Antecaridina* sp. East Timor) not displayed.

The two described *Stygiocaris* species were recovered as a clade. A third *Stygiocaris* taxon was only found at site C-28 (Bundera Sinkhole). The three species are ∼13% divergent from each other at COI (∼5% at 16S)(uncorrected). The relationship between the three *Stygiocaris* species is not clear in the two 16S datasets (Figs. 4, 6), and so we also sequenced representatives of the major intraspecific groupings within each of the three species for the conserved nuclear H3 gene and the more rapidly evolving mitochondrial COI gene. This *Stygiocaris* combined dataset unequivocally recovers *S. lancifera* and *S. stylifera* as sister taxa relative to *Stygiocaris* sp. Bundera in each individual gene analysis and when the three analyses are combined (Fig. 7).

### Intraspecific groupings within *Stygiocaris* species

There is significant intraspecfic diversity within *S. stylifera* and *S. lancifera* visible in the *Stygiocaris* 16S, COI and combined datasets (Figs. 6, 7). Within *S. stylifera* there are three subspecific groups, which are ∼6% divergent from each other in their COI sequences (∼2% 16S). One of these three groups was only found on the eastern side of the Cape Range in the Defence Bores. The second group was only found at C-25 on the western side of the Cape, and the third group at both C-25 and Barrow Island.

**Figure 8:**
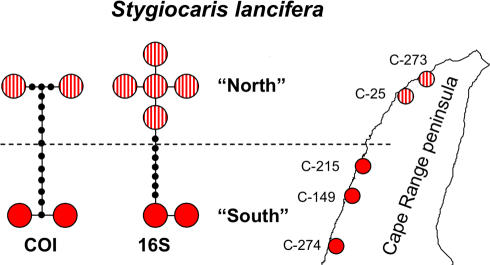
COI and 16S haplotype networks for *Stygiocaris lancifera* placed in Cape Range geographic context.

There were two groups within *S. lancifera* which are ∼2% divergent at COI (∼1% 16S). These groups are geographically structured (Fig. 8), with a southern (C-215, C-149, C-274) and northern group (C-273, C-25).

### Rates of molecular evolution and divergence estimates

There is a range of molecular clock calculations available, depending on whether one uses more modern relaxed clock methods or fairly simplistic distance estimates, which will vary based on whether one uses model-corrected or uncorrected distances for either COI or 16S sequences. When we marry the distance estimates with the geological estimates, we can derive rates of molecular evolution for particular nodes. These give a wide range of possible divergence rates per million years (±S.E.) for the *S. lancifera/stylifera* node (COI: 1.33–5.16%; 16S: 0.55–1.03%). Applying these rates, the common ancestor of *S. lancifera/stylifera* and *Stygiocaris* sp. Bundera diverged 6.15–12.30 MYA (±S.E.) for COI and 9.39–16.61 MYA for 16S.

Using a relaxed clock method on the Atyid 16S dataset, the time to most recent common ancestor for *S. lancifera/stylifera*/sp. Bundera is comparable to that above, with a mean of 11.08 MYA (7–17.94 MYA for 95% highest posterior density [HPD]; effective sample size [ESS] = 1612.12). The mean tmrca for *Stygiocaris* spp./*Typhlatya* is 24.53 MYA (11.11–41.54 MYA 95% HPD; 1449.00 ESS).

The mean 16S divergence rate resulting from the relaxed method is 0.75% per million years (0.33%–1.25% for 95% HPD; 1126.55 ESS), which is also comparable to the point estimates above for the *S. lancifera/stylifera* node. Commonly used general interspecific rates for crustaceans are 0.65% [73] and 0.9% [74] for 16S and 1.25% [75] or 1.4% [76] for COI. Our interspecific COI rates are considerably slower than recently inferred intraspecific rates for populations of the related atyid (*Halocaridina*, ∼20% per million years [47]). This may be a result of hypothesized differences in rates of molecular divergence between more recent intraspecific datasets and more ancient interspecific ones (see [77], [78]).

## Discussion

### Western Australian cave atyids at the global scale

An earlier study of Australian atyids [35] has shown that some subterranean species (*Parisia unguis, Pycnsia raptor*) descend from localised surface species rather than morphologically similar subterranean species from further afield, and that formal systematic classifications often do not agree with inferred evolutionary relationships. We found a similar pattern in the present study for two further cave species (*Parisia gracilis, Pycneus moritans* Holthuis, 1986). However, importantly, we did not find this pattern for *Stygiocaris* spp., which formed no clades with any Australian taxa. *Stygiocaris* formed a clade with subterranean species (largely from the subfamily Typhlatyinae, Fig. 4) found over a very large area, thus refuting our hypothesis that *Stygiocaris* would follow a pattern similar to other Australian subterranean atyids.

Interestingly the nearest relation to *Stygiocaris* in both our mitochondrial and nuclear data is the subterranean genus *Typhlatya*, whose centre of diversity is the Caribbean/North Atlantic/Mediterranean [40]. This pattern fits closely with the hypothesis that certain widespread disjunct anchialine species have descended from marine species from the Tethys Sea, whose disjuncture can be explained by sea floor spreading due to plate tectonic movement [27], [30], [51]. The severing of migration routes caused by the closure of the Tethys Seaway about 19 MYA cut the link between the previously closely related marine faunas on either side [31], [79]. Our mean estimate of a common ancestor of *Stygiocaris* and *Typhlatya* at 24.53 MYA (11.11–41.54 MYA) is congruent with a “Tethyan Track” explanation [80].

*Stygiocaris* is by no means the only Cape Range representative of this pattern, as the Bundera Sinkhole (site C-28) hosts species of remipede crustaceans [28], thaumatocypridid ostrocods [81] and certain copepods [80] (and the thermosbaenacean genus *Halosbaena* from C-215, Poore & Humphreys 1992) found nowhere else in the Southern Hemisphere, whose sister taxa are instead in coastal caves of the North Atlantic (Caribbean Islands, Yucatán, Bermuda, Canary Islands) [26].

One interesting point is that the closure of the Tethys Seaway (19–10 million years [31], [32]) is older than the formation of the Cape Range (10–7 million years [14], [21]). This is likely explained by either 1) the ancestors of *Stygiocaris* colonising the coast of the nearby coast of Pilbara Craton first and later moving to the Cape Range area [17], [27], or 2) local marine species actively colonising or being stranded in newly emergent limestone habitat (“regression”; [82]) and their marine ancestors becoming extinct [36] or remaining unsampled [34]. A similar scenario to the second explanation is envisioned for the *Stygiocaris*'s sister taxon, *Typhlatya*, that are hypothesised to have lived in marine caves in the Caribbean before the formation of their current anchialine habitats [40].

A true evolutionary history of any species is unlikely to conform to a single, simple idea, such as Tethyan vicariance, and a more complex “mixed-model” approach is likely to better reflect reality, as found in many studies of subterranean crustaceans [9], [75] . The *Stygiocaris/Typhlatya* relationship on its own is only weak evidence for an imprint of the Tethys, especially as other atyids have proven to disperse over great distances [35], [36], [48], [49], but similar “Tethyan” patterns amongst sympatric taxa imply that this idea must be considered at least feasible at the large scale. This will be testable as more molecular data from sympatric taxa becomes available [41]. It is also likely a question of scale [37], [48], with Tethyan vicariance responsible for some global distributions, but overlaid with both long and short distance dispersal and vicariance [37], [40], [51].

### Diversity of *Stygiocaris* species

If the evolutionary relatives of the genus *Stygiocaris* lie in a far off land, there is no doubt that the various *Stygiocaris* species themselves are endemic to northwestern Australia. The two described *Stygiocaris* species are largely found on opposite sides of the Cape Range, with *S. lancifera* on the western side and *S. stylifera* on the east and Barrow Island, with a few sites of sympatry in the northwest of the peninsula (Fig. 2) [18]. Both species are highly troglobitic (transparent, reduced eyes, <20mm [38]; Fig. 3b). The two species are difficult to distinguish from each other [18], [38], [52], [71], but their evolutionary differentiation has been confirmed using allozymes [18], [19], DNA (present study) and recent morphological work (WFH and Dr Danny Tang).

The east/west split between species may be the result of isolation by the orogeny of the range, with limited secondary contact in the northwest, probably during epochs of lowered sea-levels [17]. Secondary contact also likely explains the sympatry of some *Typhlatya* species in Mexico [40], [45] and ostrocod species of the genus *Danielopolina* Kornicker & Sohn, 1976 in the Canary Islands [81] . The sympatric blind gudgeon *Milyeringa veritas* Whitley, 1945 also shows a split between east and west with a site of overlap in the north [18], [19]. This divergence is at a smaller, intraspecific level, which may also imply a persistent dispersal barrier near the northern tip of the peninsula [18], limiting dispersal between east and west for most stygofauna.

Our molecular data suggest the presence of a third, cryptic, species of *Stygiocaris* at the Bundera Sinkhole (site C-28, Fig. 2). There is a proliferation of cryptic species in subterranean habitats both because of limited study due to difficult access and rampant morphological convergence in the face of strong, similar selective pressures [7], [8], [43]. Cryptic species have been identified using molecular methods within other subterranean taxa in the Cape Range (millipedes [20], amphipods [19]) and Western Shield (amphipods [5], [6], Parabathynellidae [16]) and within other subterranean atyids (*Typhlatya* [40], [45]). This has meant that widespread groundwater “species” have proven to be complexes of cryptic species [11], highly compartmentalised by their underground landscapes [7]. As for *Stygiocaris* sp. Bundera, these speciations have often been associated with very limited or undetected morphological differentiation [20]. Species discovered with molecular methods can help to define species boundaries and foster further, targeted morphological study, which can go on to discover new morphological characters [43].

The *Stygiocaris* specimens from Bundera were originally identified as *S. stylifera* [52], but there has always been some doubt (WFH) due to their large size, Bundera's isolation from other *Stygiocaris* sites (>30 kms, Fig. 2), and the very high salinity water in which they occur (20,000–35,000 mg L^−1 ^; [28]), whereas other *Stygiocaris* species are only in fresh to brackish water (mean = 2064 mg L^−1^ TDS, sd 1753; range 290–7700, n = 26) [52]. Bundera Cenote (sinkhole) is of particular interest as it is the only deep anchialine system in Australia (penetrating down to the underlying seawater) and the only continental one in the Southern Hemisphere [28]. The water profile at Bundera is highly stratified, with warm sea water beneath a stable thermo-halocline [28]. It contains *Stygiocaris* sp. Bundera (as well as the unique “Tethyan” community referred to above), which were only sampled well beneath the overlaying layer of brackish water, in water with a salinity similar to local seawater [28]. This occurrence of different congeneric species in distinct physiochemical conditions is also found within *Stygiocaris*'s sister, *Typhlatya*, which hosts both fully freshwater and brackish species [40], and copepods in European karst [37].

The brackish-seawater environment of *Stygiocaris* sp. Bundera may well represent the original lifestyle of the coastal marine Tethyan ancestor of *Stygiocaris* and *Typhlatya*, with some species subsequently invading and adapting to freshwater. The dispersal of freshwater species is limited by saline conditions, whereas *Stygiocari*s sp. Bundera may be limited by surrounding freshwater. There is considerable evidence, from the nature of the faunas and their distribution, that anchialine habitats are both geologically very old and persistent [1], [25], [83] so providing a potentially persistent “Tethyan” time capsule of which Bundera is the present manifestation. These anchialine systems do not necessarily need to rely on allochthonous energy sources. Food web studies using stable isotopes of *Stygiocaris* (at Bundera [28] and Barrow Island [24]) and *Typhlatya* [42] have suggested and demonstrated respectively that they feed on sulphide-oxidising chemoautotrophic bacteria (which are common in anchialine systems [24], [84]), thus making these systems at least partially independent from the surface, similar to some deep-sea vent communities [42].

### Intraspecific diversity at the local scale

Manifestations of the uplift and subsequent karst development in Cape Range are found in the subterranean fauna, both terrestrial and aquatic. The terrestrial cave fauna differs along the length of the range, and between the coast and the range, with one terrestrial species, a troglobitic micro-whipscorpion, found on the east coast plain and Barrow Island, like S*tygiocaris* [85]. *Norcapensis* Bradbury & Williams, 1997, a melitid amphipod, inhabits perched groundwater at elevations of about 200 m in Cape Range, where three distinct allozyme populations occur [18]; melitids are a marine family that has invaded inland waters and those high in Cape Range probably uplifted with the range.

There is significant geographic intraspecific biodiversity within many groundwater species, visible within both described *Stygiocaris* species (Figs. 6, 7), as well as within other Cape Range stygofauna (amphipods [19], and gudgeons [19]), which confirms our second hypothesis. In particular, the populations within the linear habitat of *S. lancifera* are split into divergent northern and southern groups (Fig. 8) at a very similar point to geographic provinces of the other taxa mentioned above. This comparative phylogeographic approach strengthens the hypothesis that common events or dispersal barriers [41], such as sea-level changes [26], gorges cutting through to impermeable limestone layers [20], or hypersaline groundwater [27], has structured and isolated many of the populations into distinct geographic communities. This has conservation implications in that we will need to avoid any human mediated exchange of individuals between evolutionary distinct populations [26].

### Conservation of groundwater communities

Our multi-scale molecular data have allowed us to identify unappreciated groundwater biodiversity at the alpha to gamma levels, ranging from localised population differences to ancient globe-spanning evolutionary relationships. Groundwater faunas generally are vulnerable to human impacts resulting variously from water extraction, the addition of substances to water, and changes to aquifer permeability. As these isolated and widely vicariant anchialine communities occur in tightly constrained coastal locations, they may be particularly vulnerable [86] or especially resilient [25], [28], [87], depending on the nature of the threat. This dichotomy of interpretation of the evidence, which needs to be addressed globally, may result from the extremely sharp gradients in physio-chemical conditions that may occur in anchialine habitats [24]. Issues potentially or actually pertinent to the Cape Range/Barrow Island groundwater fauna include oil and gas field development [24], mining [3], water extraction or various uses and waste discharge [23], increasing salinity [10], and pollution [1].

Although *Stygiocaris* is only one component of the local biota, our data show that it is both an Australian element of the ancient Tethyan fauna and harbours cryptic species with very small ranges (possibly even a single site) and thus liable to extinction [7]. Indeed, Bundera Sinkhole is listed as a specially protected community in Western Australia, and a number of the species, including *S. lancifera,* the remipede *Lasionectes exleyi* Yager & Humphreys 1996, and both species of cave fish, are specially protected under Commonwealth of Australian and/or Western Australian fauna legislation.

Subterranean habitats tend to have truncated [88], easily disrupted, food webs [26], within which *Stygiocaris*, may play a keystone role (and be a potential bioindicator [10]). This is because atyids are biofilm feeders that may act as a conduit between energy producing chemoautotrophic bacteria and vulnerable higher-trophic level vertebrate predators, such as the blind gudgeon [26]. Because the basic biology of the diverse groundwater fauna of Australia is largely unknown [13], it is not possible to assess adequately their vulnerability to anthropogenic changes. As we add more information on their evolution and ecology, the arguments for the protection of all groundwater habitats are considerably strengthened [1].
